# Sodium Nitroprusside Functions in Browning Control and Quality Maintaining of Postharvest Rambutan Fruit

**DOI:** 10.3389/fpls.2021.795671

**Published:** 2022-01-11

**Authors:** Ruining Zhang, Zhouyu Yuan, Yuwei Jiang, Fan Jiang, Ping Chen

**Affiliations:** ^1^Key Laboratory for Quality Regulation of Tropical Horticultural Plants of Hainan Province, College of Horticulture, Hainan University, Haikou, China; ^2^Sanya Nanfan Research Institute of Hainan University, Hainan Yazhou Bay Seed Laboratory, Sanya, China

**Keywords:** rambutan, sodium nitroprusside, surface browning, quality, postharvest, preservation

## Abstract

Surface browning after harvest is the primary constraint affecting the storage life and market circulation of rambutans. In this study, rambutan fruits were soaked in sodium nitroprusside at different concentrations and stored at 25°C for 8 days to explore the effects on postharvest quality and browning. The weight loss, browning index and superoxide anion radical, hydrogen peroxide and malondialdehyde contents of the treated fruits were reduced compared to those of the control fruits (soaked in distilled water). And fruits treated with sodium nitroprusside had a higher total phenolic content and lower polyphenol oxidase and peroxidase activity. In addition, compared with the control, the treated fruits exhibited higher phenylalanine ammonia lyase, ascorbate peroxidase and superoxide dismutase activities; titratable acidity; and soluble solid, vitamin C and protein contents, indicating high fruit quality. Overall, sodium nitroprusside treatment at 200 μmol L^−1^ demonstrated the most positive preservation effects. Therefore, sodium nitroprusside treatment, particularly at 200 μmol L^−1^, can be used as an eco-friendly, safe and convenient method for postharvest quality management and high-efficiency preservation of rambutan fruits.

## Introduction

Rambutan (*Nephelium lappaceum* L.), native to Southeast Asia, is a non-climacteric tropical fruit belonging to the family *Sapindaceae* (Sun et al., 2010). At present, rambutans are widely cultivated in countries, such as Thailand and Malaysia. Rambutan from Malaysia has been introduced and planted in Hainan, China and Baoting has become the best rambutan growing area in Hainan (Yang and Cao, 2005). Owing to their attractive appearance, gorgeous colour, delicious taste and rich nutritional value (Martínez-Castellanos et al., 2009), rambutans are becoming increasingly popular. However, rambutans are prone to peel browning, decay and deterioration during transportation and distribution 3 days after harvest (Shao et al., 2013), which restricts market circulation and consumer appeal (Sivakumar and Korsten, 2010), resulting in a substantial reduction in marketability and economic value (Lin et al., 2020a). Therefore, it is necessary and significant to adopt certain technical methods to slow down the browning of rambutans to ensure postharvest quality and an extended shelf life.

In general, the browning of fruits and vegetables is related to tissue damages including mechanical damage and pathogen infection, as well as biochemical reactions including membrane lipid peroxidation, active oxygen metabolism and enzymatic reactions of phenolic compounds (Jiang et al., 2004; Degl’Innocenti et al., 2007; Maneenuam et al., 2007; Sajid et al., 2019a). Research has been conducted on postharvest browning and the preservation of rambutans. Shao et al. (2013) and Lin et al. (2020a,b) studied the effects of different packaging materials and temperatures on the quality and browning of postharvest rambutan fruit. Hernández-Arenas et al. (2012), Cui et al. (2015) and Chen et al. (2016) have determined that different temperatures combined with modified atmospheres and vacuum treatments improve the postharvest quality of rambutans and prolong their storage life. Li et al. (2018, 2019b) investigated the functions of several plant essential oils in the preservation of rambutans after harvest. In addition, research using a combination of chemical agents and biocontrol agents has been conducted to explore the browning and quality changes of postharvest rambutans (Martínez-Castellanos et al., 2009; Ahmad et al., 2017).

Research has shown that, as a regulatory factor of plant growth and development (Badiyan et al., 2004), an appropriate concentration of nitric oxide can inhibit ethylene release, reduce respiration rate, hinder Acetyl-CoA carboxylase synthesis and slow down the process of plant senescence by regulating ethylene metabolism (Zhu and Zhou, 2007; Jiang et al., 2010). Consequently, nitric oxide can be used to inhibit the browning and quality deterioration of horticultural products after harvest. Some studies have explored the application of nitric oxide in the preservation of climacteric fruits, such as the banana (Wang et al., 2015), as well as non-climacteric fruits, such as strawberry (Zhang et al., 2014) and longan (Duan et al., 2007). Informed by these previous studies, we explored the postharvest preservation and browning of rambutan fruit using sodium nitroprusside, a source of exogenous nitric oxide.

We selected sodium nitroprusside for experimentation on rambutan, a characteristic non-climacteric tropical fruit, because this chemical has no residual toxicity to fruits and is a cost-effective means of fruit preservation (Zhu et al., 2008). A preliminary study was conducted on the preservation effects of different concentrations of sodium nitroprusside. In subsequent experiments, the concentrations of sodium nitroprusside were determined, and the effects of this chemical on postharvest quality, browning, active oxygen metabolism and the antioxidant system of rambutan fruit were studied. As a new, safe and low-cost preservative, sodium nitroprusside is rarely used to treat postharvest tropical fruits, and there are no published reports on the application of sodium nitroprusside for preservation of rambutan fruit. In addition, nitric oxide derived from sodium nitroprusside is known to have a more significant preservation effect on non-climacteric fruits, such as rambutan compared with climacteric fruits (Leshem, 2000). The use of sodium nitroprusside as a rambutan fruit preservation agent represents a potential innovation as there are no associated safety hazards, and the resultant nitric oxide can inhibit the quality deterioration and enzymatic browning process, thereby extending the shelf life and improving the commercial value.

## Materials and Methods

### Materials

The rambutan fruits (Baoyan No. 7) used in this study were picked on the same day in an orchard in Baoting, Hainan, China. The experimental fruits were basically selected with 80% maturity and uniform of shape, size and colour; simultaneously, there was no disease or mechanical injuries. In addition, bump damage during transportation of the fruits from the orchard was minimised. The sodium nitroprusside (AR, relative molecular mass 297.95) used in this study was manufactured by the Tianjin Beichen Fangzheng Reagent Factory (Tianjin, China).

### Treatments

A total of 576 fruits were tested during the experiment (4 treatments × 18 fruits in each group × 8 sampling intervals). The four groups of rambutans were equally separated, and the carpopodiums of fruits were kept at 0.5 cm in length. The fruits were then soaked in a prepared sodium hypochlorite solution (0.1 Â mL L^−1^) for 2 min and 0.5 g L^−1^ SporGon solution (Decon Labs, Inc., King of Prussia, PA, United States) for 5 min for sterilisation and disinfection. Subsequently, they were rinsed using distilled water. Fruits from one of the four treatment groups were soaked in distilled water as a control (treatment CK), whereas fruits of the other three groups were soaked in sodium nitroprusside solution at concentrations of 100 μmol L^−1^, 200 μmol L^−1^ and 300 μmol L^−1^, representing treatments A, B and C, respectively. All treatment group fruits were immersed in sodium nitroprusside for 2 h. Thereafter, the fruits were air-dried in a ventilated indoor area and then stored in perforated polyethylene bags (each hole with Ø 0.6 mm, 20 μm thick) 25°C for a period of 8 days. Fruits were sampled every day during the storage period, and a composite sample of 18 fruits was assayed each time. Of the 18 fruits, 6 were defined as a technical replicate and biological replicate repeated three times. The weight loss, browning index, firmness, total soluble solid (TSS) content and titratable acidity (TA) of the fruits were evaluated on each sampling day. For determination of vitamin C content, total phenolic content (TPC), reactive oxygen metabolism and enzyme assays, the daily samples were preserved at −80°C until assayed. The peel was used to assay browning index, firmness, malondialdehyde (MDA) content, reactive oxygen species and phenolic metabolism indexes. The pulp was used for TSS, TA, vitamin C and protein content determination, and weight loss was determined using the whole fruit.

### Determination of Browning Index, Weight Loss and Firmness

Browning was judged visually, and fruits were allocated to a browning level as follows: no pericarp browning = 0; 0–25% pericarp browning of the whole fruit = 1; 25–50% pericarp browning = 2; 50–75% pericarp browning = 3; 75–100% pericarp browning = 4 (Shao et al., 2013). The browning index of rambutan fruits was calculated as follows:


$$\text{Browning}\;\;\text{index}=\frac{\Sigma \left(\text{Browning}\;\;\text{level}\times \text{Number}\;\;\text{of}\;\;\text{fruits}\;\;\text{in}\;\;\text{each}\;\;\text{level}\right)}{\text{Maximum}\;\;\text{level}\times \text{Total}\;\;\text{number}\;\;\text{of}\;\;\text{fruits}\;\;\text{in}\;\;\text{each}\;\;\text{treatment}}$$

Weight loss was determined by weighing the fruits on an electronic balance prior to storage and at the end of every sampling interval. Weight loss was calculated and expressed as a percentage.

Firmness was measured directly using a durometer (GY-4; Top Cloud-Agri Technology Co., Ltd., Zhejiang, China). An average of three points was selected around the equatorial plane of the fruits for firmness determination and repeated three times (Ahmad et al., 2017; Li et al., 2018).

### Determination of MDA Content

The MDA content was determined following the procedure of Zou (2000) and Yang et al. (2011) with a slight modification. Peel (0.5 g) was ground into a homogenate with 5 ml of 5% (w/v) trichloroacetic acid and then centrifuged. A total of 2 ml aliquots each of the supernatant and trichloroacetic acid solution were mixed, and the resultant solution was boiled at 95°C for 30 min. The mixed solution was then cooled and centrifuged at 3,000 × *g* for 10 min, and the absorbance was determined at 600 nm, 532 nm and 450 nm using a visible spectrophotometer (UV-Vis 721; Shanghai Yoke Instrument Co., Ltd., Shanghai, China). The MDA content was calculated and reported in μmol g^−1^.

### Assay of ${\text{O}}_{2}^{{-}^{\cdot }}$ and H_2_O_2_ Contents

The methodology of Zhu (2013) and Cao et al. (2019) was used to determine the ${\text{O}}_{2}^{{-}^{\cdot }}$ content. Peel samples (0.5 g) were homogenised in extraction buffer (5 ml) containing 1 mmol L^−1^ EDTA, 0.3% Triton X-100 and 2% polyvinylpyrrolidone, followed by centrifugation at 12,000 × *g* for 20 min. The supernatant (1 ml) was mixed with 1 ml of phosphate buffer (50 mmol L^−1^, pH 7.8) and hydroxylamine hydrochloride (1 mmol L^−1^) and maintained at 25°C for 1 h. Thereafter, 1 ml of 4-aminoben zenesulfonic acid solution (17 mmol L^−1^) and 1 ml α-naphthylamine solution (7 mmol L^−1^) was added to the mixture. The absorbance was determined at 530 nm after 20 min, and a standard curve was developed using potassium nitrite. The ${\text{O}}_{2}^{{-}^{\cdot }}$ content was reported in μmol min^−1^ g^−1^.

The H_2_O_2_ content was determined using a Nanjing Jiancheng Bioengineering Institute kit (Bioengineering Inc., Nanjing, China). Peel (0.2 g) was ground with 1.8 ml of phosphate buffer (pH 7.0) and centrifuged to take the supernatant. After mixing with the reaction reagents, the absorbance was measured at 405 nm and reported in mol g^−1^ in accordance with the reaction with molybdic acid.

### Determination of TPC

Following the Folin-Ciocalteu method (Choi et al., 2011; Niu et al., 2020), the TPC in the peel was analysed. Peel tissue (0.5 g) was ground into absolute ethyl alcohol and extracted for 2 h at 4°C in the dark. After centrifugation at 12000 rpm, the supernatant was mixed with Foline-Phenol reagent and sodium carbonate solution (10%). The mixture was kept at 50°C for 30 min, and the change in absorbance at 700 nm was measured. The standard curve was constructed using gallic acid as the standard substance, and the content was reported in μg g^−1^.

### Assay of Enzyme Activities

Following the kit instructions (Nanjing Jiancheng Bioengineering Institute), the polyphenol oxidase (PPO) activity was assayed. Peel (0.2 g) was ground with 1 ml of extract reagent and centrifuged at 8000 rpm for 10 min. The absorbance was determined at 420 nm. Enzyme activity leading to an absorbance change of 0.01 units per minute in each millilitre of solution was defined as one enzyme activity unit (U).

Peroxidase (POD) activity, based on the principle of the H_2_O_2_ catalytic reaction, was determined using Nanjing Jiancheng Bioengineering Institute kits. Peel sample (0.2 g) was ground with phosphate buffer (pH 7.0) and vortex extracted for 3 min. The mixture was centrifuged at 4000 rpm for 20 min and the absorbance of the supernatant was determined at 420 nm. One POD U was equivalent to the quantity of enzyme that catalysed 1 μg of substrate per minute.

Phenylalanine ammonia lyase (PAL) enzyme activity was assayed according to Yingsanga et al. (2008) and Cao et al. (2019), with modifications. Peel (0.5 g) was ground in extraction buffer (5 ml) and centrifuged at 12000 × *g* for 30 min. The supernatant (0.5 ml), 50 mmol L^−1^ boric acid buffer (3 ml) and 20 mmol L^−1^ l-phenylalanine solution (0.5 ml) were reacted, and the absorbance was determined at 290 nm using a UV-VIS spectrophotometer (T6; Beijing Purkinje General Instrument Co., Ltd., Beijing, China). One PAL U represented the enzyme activity that increased the absorbance by 0.01 of a unit.

As ascorbic acid reacts with hydrogen peroxide, ascorbate peroxidase (APX) activity was according to kits from Nanjing Jiancheng Bioengineering Institute. The peel samples (0.2 g) were ground with the buffer solution and centrifuged at 10,000 rpm for 10 min. The supernatant, buffer solution, substrate solution and matrix solution were mixed, and the absorbance was measured at 290 nm. One U indicated the quantity of enzyme oxidising 1 μmol of ascorbic acid per minute in each millilitre of solution.

Superoxide dismutase (SOD) activity was evaluated using Nanjing Jiancheng Bioengineering Institute kits. Peel (0.2 g) was ground into a homogenate in phosphate buffer (pH 7.0) and then centrifuged for 10 min (4,000 rpm). The supernatant and the reaction solution were vortex mixed and kept at 37°C for 40 min. The mixture was reacted with the chromogenic agent, and the absorbance change was measured at 550 nm. One U was equivalent to the amount of enzyme corresponding to the 50% inhibition rate of enzyme in each millilitre of reaction solution.

### Determination of TSS and TA Contents

TSS content was determined using a digital refractometer (PAL-1; ATAGO Co., Ltd., Japan) and reported as a percentage. Following the procedure of Long and He (2002) with a slight modification, the TA content was assayed with NaOH (0.01 mol L^−1^). Pulp (3.0 g) was ground into distilled water (50 ml) and heated in water bath for 30 min (80°C). The supernatant was used for titration with sodium hydroxide solution. The TA content was calculated based on the consumption volume of sodium hydroxide and reported as a percentage.

### Determination of Vitamin C and Protein Contents

The vitamin C content in rambutan fruits was determined using kits provided by Nanjing Jiancheng Bioengineering Institute. Pulp sample (0.2 g) was ground into phosphate buffer (pH 7.0), mixed with the reaction reagent and centrifuged at 4000 rpm for 10 min. The supernatant was reacted with three application solutions and kept at 37°C for 30 min. The mixture was merged in the reaction reagent to determine the absorbance at 536 nm according to the principle of Fe^3+^ reduction. Vitamin C content was calculated and expressed in g kg^−1^.

According to Nanjing Jiancheng Bioengineering Institute kits, the protein content in the fruit pulp was assayed using the Bradford method (Bradford, 1976). Pulp tissue (0.2 g) was ground with phosphate buffer (pH 7.0) and centrifuged at 4000 rpm for 10 min. The supernatant was reacted with coomassie brilliant blue solution for 10 min, and the absorbance was determined at 595 nm. Distilled water instead of the sample was considered a control, and the content was presented as gprot L^−1^.

### Data Analysis

The research was designed, and the data were analysed using a completely randomised design. Analysis of variance (ANOVA) was performed for statistical analysis, and different parameters were evaluated using Duncan’s multiple range test (*p* < 0.05) using SAS 9.1 software.

## Results

### Browning, Weight Loss and Firmness

**Figure 1:**
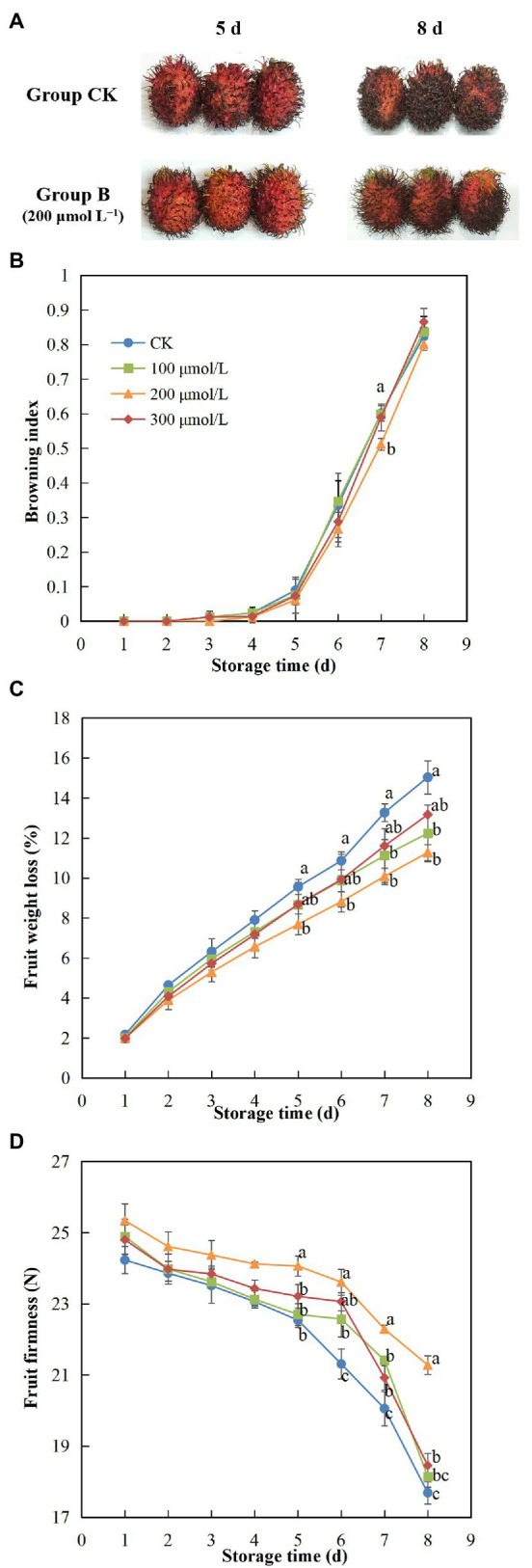
Effect of sodium nitroprusside at different concentrations on the browning **(A,B)**, weight loss **(C)**, and firmness **(D)** of rambutan fruits stored at 25°C for 8 days. Data are the average of three replicates. Vertical bars represent standard deviations, and different letters indicate significant differences (*p* < 0.05).

During storage, the rambutan fruits tended to brown (Figure 1A), especially the browning of group B (200 μmol L^−1^ sodium nitroprusside) was more obvious than control. Also, the browning index of the control fruits showed the most rapid increase, whereas the fruits in treatment group B had the lowest rate of browning (Figure 1B). This significant reduction in browning rate in response to treatment with sodium nitroprusside supports the use of this chemical for increased postharvest storage and distribution of rambutan fruits. The fruits in the control group exhibited significantly higher weight loss compared to those soaked in sodium nitroprusside (Figure 1C), and the inhibitory effect on weight loss of the 200 μmol L^−1^ treatment was the most obvious. After 8 days of storage, the weight loss of group B fruits was 20% lower than that of the control group fruits. In addition, a decrease in fruit firmness was suppressed by sodium nitroprusside, especially in the fruits treated with 200 μmol L^−1^, which maintained the greatest fruit firmness of all groups over the storage period (Figure 1D). The firmness of the control group fruits declined sharply on day 6, whereas a similar sharp decline in the firmness of fruits treated with sodium nitroprusside occurred from day 7, indicating a delay in fruit softening of 1 day. Thus, sodium nitroprusside appears to have an inhibitory effect on drastic changes in fruit firmness, which could contribute to maintaining the quality and freshness of harvested fruit. On day 8, the fruits of treatment B were the firmest fruits in all treatments, and the average firmness of treatment B fruits at the end of the storage period was approximately 1.2 times greater compared with the control group fruits.

### MDA Content

**Figure 2:**
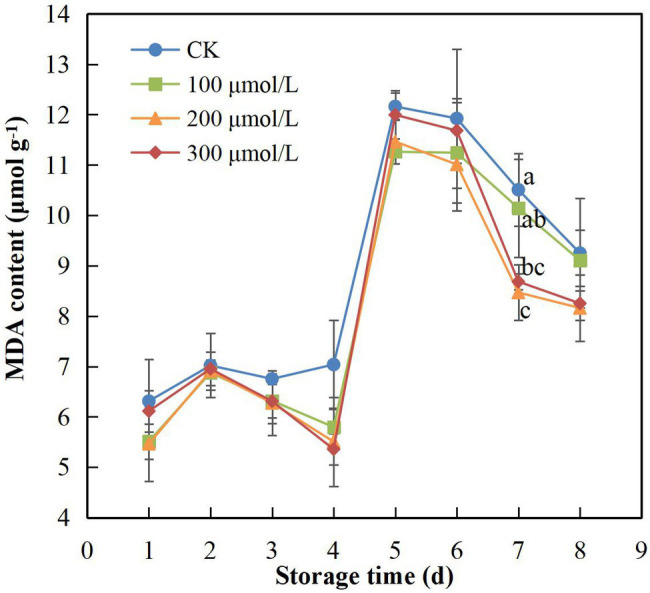
Effect of sodium nitroprusside at different concentrations on the malondialdehyde content in the peel of rambutan fruits stored at 25°C for 8 days. Data are the average of three replicates. Vertical bars represent standard deviations, and different letters indicate significant differences (*p* < 0.05).

Compared with the control, sodium nitroprusside significantly hindered the production and accumulation of MDA after harvest (*p* < 0.05; Figure 2). The MDA content of rambutan fruits treated at 200 μmol L^−1^ was maintained at the lowest level compared with the other treatments. Over the storage period, the MDA content in the control fruits was higher compared to the MDA content in all the treatment groups, indicating that sodium nitroprusside had an inhibitory effect on the production of MDA. On day 7, the MDA content in fruits in the control group differed significantly from that in the fruits in treatment groups B and C (Figure 2).

### ${\text{O}}_{2}^{{-}^{\cdot }}$ and H_2_O_2_ Contents

**Figure 3:**
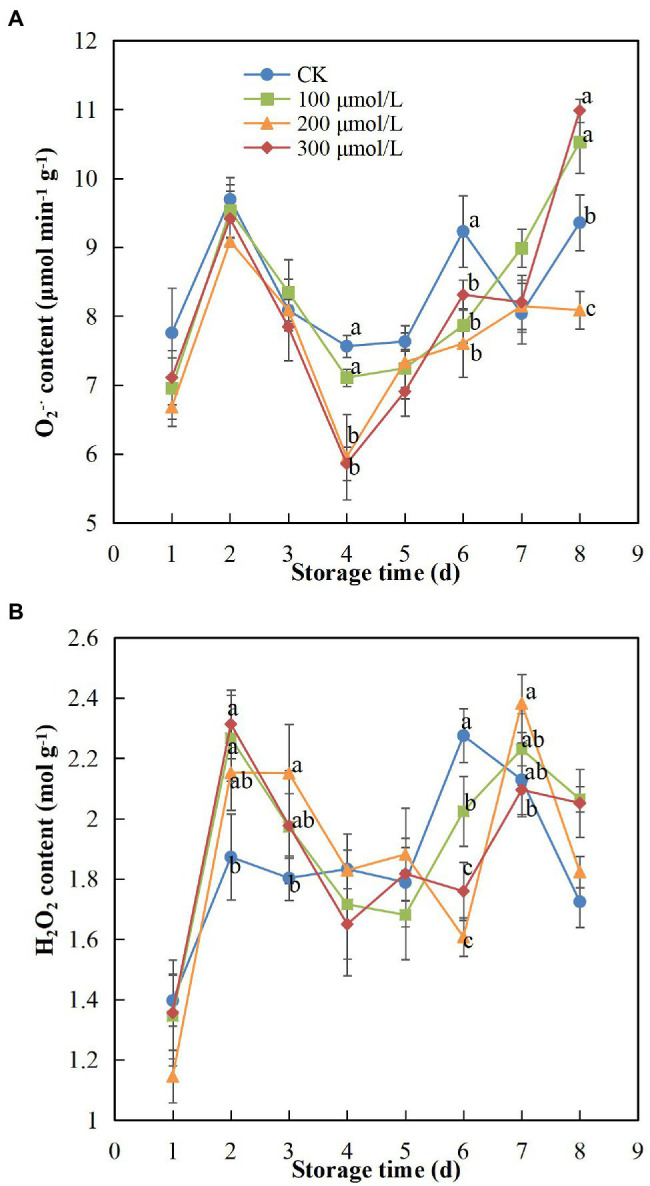
Effect of sodium nitroprusside at different concentrations on the O_2_^–•^ **(A)** and H_2_O_2_ **(B)** contents in the peel of rambutan fruits stored at 25°C for 8 days. Data are the average of three replicates. Vertical bars represent standard deviations, and different letters indicate significant differences (*p* < 0.05).

During storage, the contents of ${\text{O}}_{2}^{{-}^{\cdot }}$ and H_2_O_2_ in the peel tended to increase initially, then decrease and then increase again (Figure 3). Sodium nitroprusside appeared to inhibit the production of ${\text{O}}_{2}^{{-}^{\cdot }}$ as treatment B maintained the lowest ${\text{O}}_{2}^{{-}^{\cdot }}$ content in fruits, which was significantly different compared to the ${\text{O}}_{2}^{{-}^{\cdot }}$ content in the control fruits (*p* < 0.05). On day 4 of the storage period, the ${\text{O}}_{2}^{{-}^{\cdot }}$ content in group B fruits decreased by 1.23 times compared to that in the control fruits (Figure 3A). The H_2_O_2_ content remained at a relatively moderate level in the treatment groups; however, a sudden increase in H_2_O_2_ content of fruits treated with sodium nitroprusside occurred on day 7, showing a delay of 1 day in comparison to the control group and indicating that sodium nitroprusside likely had the effect of delaying oxidative damage to a later stage of storage. On day 6, the H_2_O_2_ content in group B fruits was more than 1.4 times lower compared to that in the control fruits (Figure 3B).

### Total Phenolic Content

**Figure 4:**
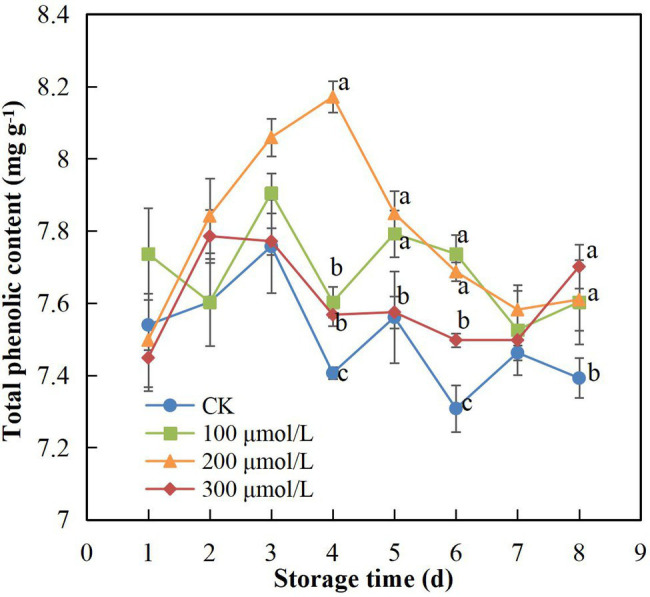
Effect of sodium nitroprusside at different concentrations on the total phenolic content in the peel of rambutan fruits stored at 25°C for 8 days. Data are the average of three replicates. Vertical bars represent standard deviations, and different letters indicate significant differences (*p* < 0.05).

During the 8-d storage period, TPC showed an overall tendency to first increase and then decrease (Figure 4). The application of sodium nitroprusside appeared to maintain the TPC in the peel at a higher level. The group B fruits (soaked in 200 μmol L^−1^) maintained the highest TPC, and the decrease appeared on day 5, which was 1 day later than the decrease recorded in the control group fruit. On day 4, the TPC of treatment B fruits was 1.1-fold higher than that of the control fruits (Figure 4).

### PPO, POD and PAL Activities

**Figure 5:**
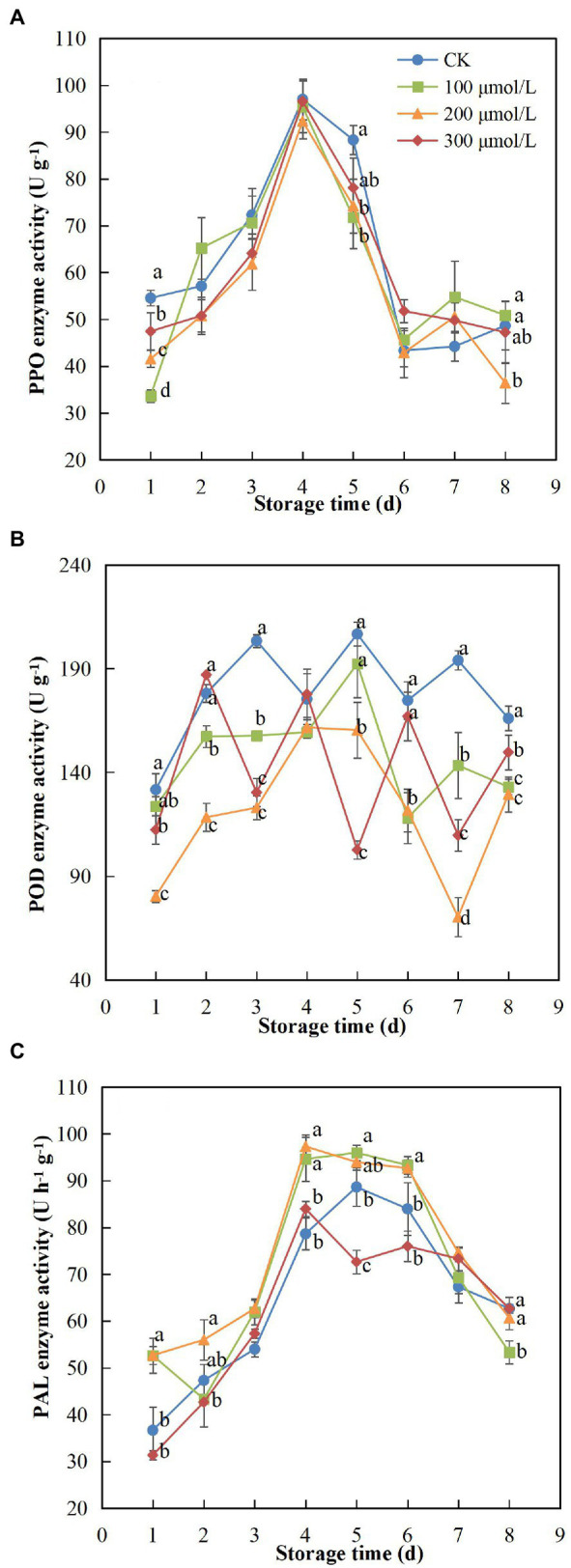
Effect of sodium nitroprusside at different concentrations on PPO **(A)**, POD **(B)** and PAL **(C)** activity in the peel of rambutan fruits stored at 25°C for 8 days. Data are the average of three replicates. Vertical bars represent standard deviations, and different letters indicate significant differences (*p* < 0.05).

PPO activity increased at the start of the storage period, peaked on day 4 and then declined until day 8 (Figure 5A). Compared to the untreated fruits, the PPO activity of fruits treated with sodium nitroprusside was relatively low, and in group B fruits (treated with 200 μmol L^−1^), the PPO activity was maintained at the lowest level, significantly different from that in the control fruits (*p* < 0.05). The activity of POD was significantly reduced after soaking in sodium nitroprusside (Figure 5B) compared to that in the unsoaked fruits (*p* < 0.05). Similar to PPO, POD activity was lowest in the 200 μmol L^−1^ treatment group during the 8-d storage period. On days 3 and 7, the POD activity in the control group fruits was 1.65 and 2.77 times higher than that in treatment group B fruits, respectively. Thus, PPO and POD activities were significantly inhibited in rambutan fruits after sodium nitroprusside treatment.

PAL activity showed a tendency to increase and then decrease, reaching a peak on 4th or 5th day (Figure 5C). In general, enzyme activity was higher in treatments A, B and C, compared to that in the control group fruits. Fruits treated with 200 μmol L^−1^ sodium nitroprusside showed the best performance, maintaining the highest PAL activity. On day 4 of storage, the enzyme activity of fruits in this treatment group was 1.24 times higher than that in the control group fruits.

### APX and SOD Activities

**Figure 6:**
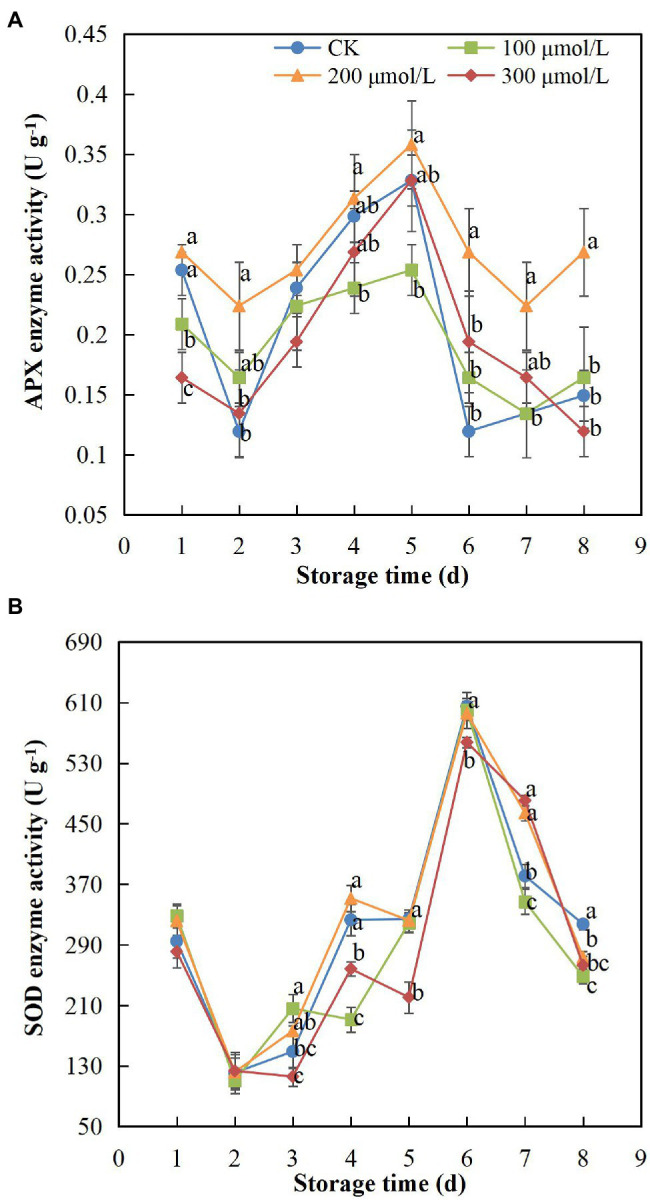
Effect of sodium nitroprusside at different concentrations on APX **(A)** and SOD **(B)** activity in the peel of rambutan fruits stored at 25°C for 8 days. Data are the average of three replicates. Vertical bars represent standard deviations, and different letters indicate significant differences (*p* < 0.05).

APX and SOD activity showed the same trend, increasing at first and then decreasing during the storage period (Figure 6). The activity of APX in treatment group B fruits remained the highest, whereas the activity change rate of control group fruits was markedly higher than that of the other groups (Figure 6A). The APX activity peaked on day 5 and began to decrease on day 6; however, the enzyme activity in group B fruits was still approximately 2-fold higher than that in the control fruits. Thus, the decline in APX activity was hindered by sodium nitroprusside (*p* < 0.05), with the treatment group B concentration showing the best effect. In addition, the SOD activity of fruits in treatment group B was higher than that of the control group fruits during storage (Figure 6B). The SOD activity decreased in all treatment groups after peaking on day 6. The enzyme activity of group B fruits was 1.22-fold higher than that of the control group fruits, indicating that a sodium nitroprusside concentration of 200 μmol L^−1^ had some delaying effect on the decrease in SOD activity.

### TSS and TA

**Figure 7:**
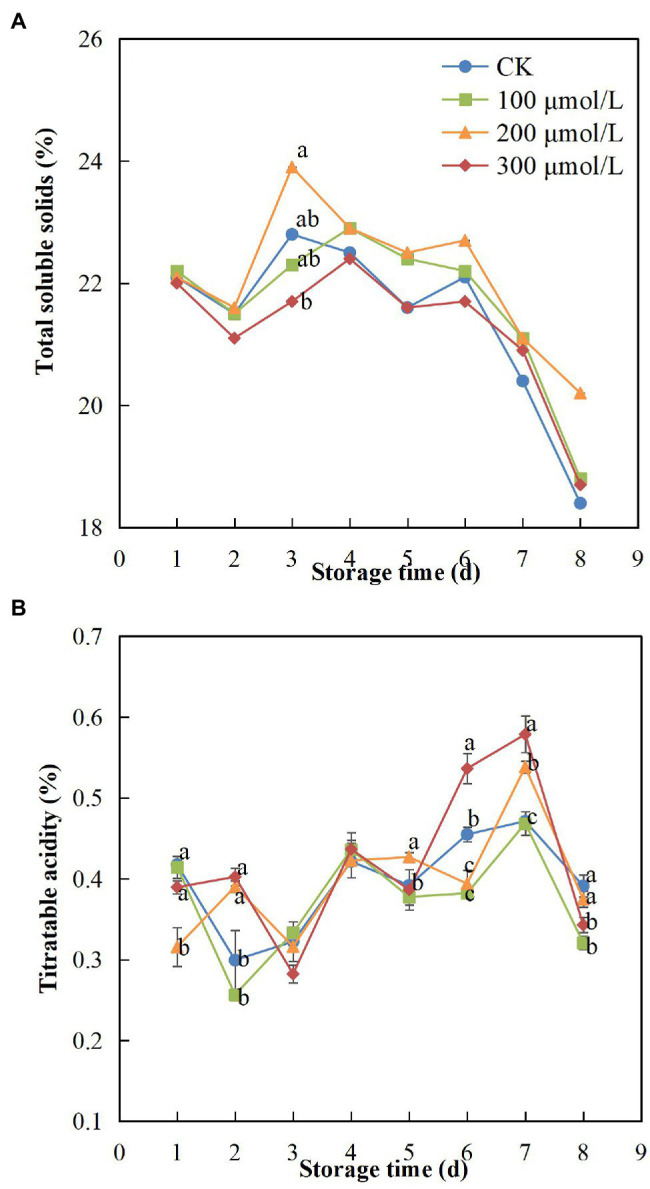
Effect of sodium nitroprusside at different concentrations on total soluble solid content **(A)** and titratable acidity **(B)** in the pulp of rambutan fruits stored at 25°C for 8 days. Data are the average of three replicates. Vertical bars represent standard deviations, and different letters indicate significant differences (*p* < 0.05).

During the experimental fruit storage period, the TSS content exhibited a tendency to increase and then decrease. The content may have increased initially because the fruits were not fully ripe at the early storage stage and then decreased after full ripeness was reached. However, compared with the control fruits, the groups treated with sodium nitroprusside showed smaller changes in TSS content, and the TSS content of treatment group B fruits remained the highest (Figure 7A). After the 8-d storage period, the TSS content in the treatment group B fruits was 1.1 times greater than that in the control fruits.

As the storage time increased, TA content of each treatment group decreased during the first 3 days, then gradually increased and then decreased rapidly after peaking on day 7 (Figure 7B). However, Chen et al. (2019) found different results, probably due to the gradual maturity of rambutan fruits over the first 3 days, which led to a decrease in TA content, following which TA content increased after the fruits ripened fully. After 7 days, the browning and decay of the fruits might have increased, resulting in a decrease in TA content. Compared with other treatments, treatment group B fruits showed minimal changes in TA, indicating that the sodium nitroprusside treatment at 200 μmol L^−1^ had a positive effect.

### Vitamin C and Protein Contents

**Figure 8:**
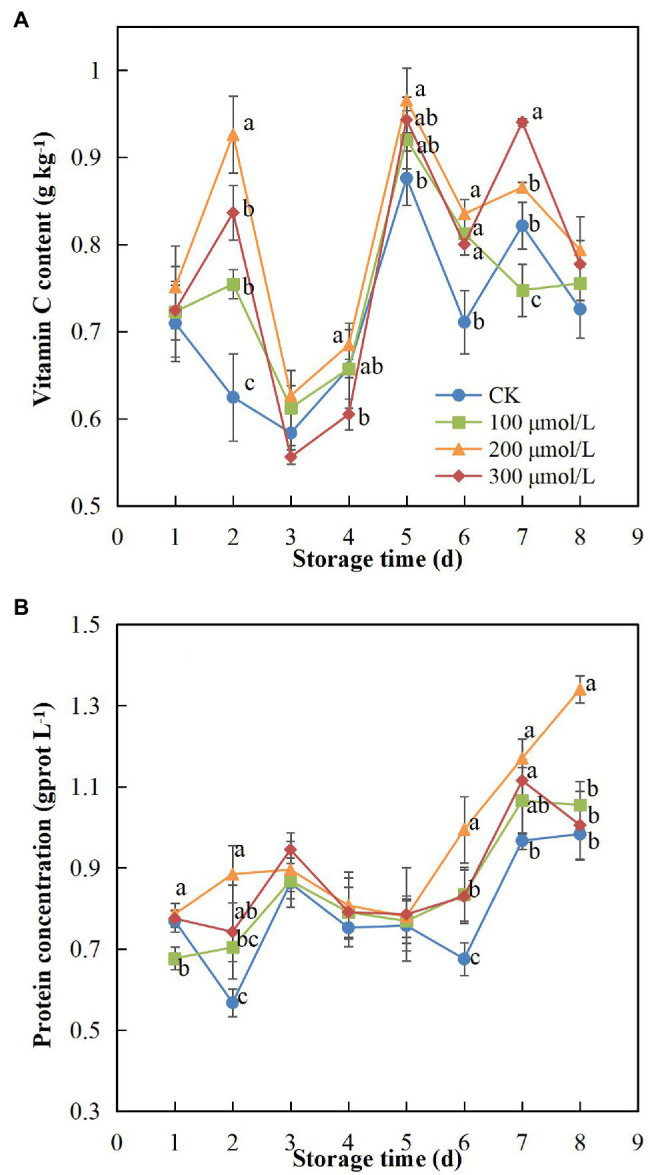
Effect of sodium nitroprusside at different concentrations on vitamin C **(A)** and protein **(B)** contents in the pulp of rambutan fruits stored at 25°C for 8 days. Data are the average of three replicates. Vertical bars represent standard deviations, and different letters indicate significant differences (*p* < 0.05).

The vitamin C content in all treatments increased initially and then decreased over the first 3 days and showed the same trend again after 5 days. The rate of increase in the treatment group fruits was higher than that in the control group fruits (Figure 8A), indicating that sodium nitroprusside had an effect on the change in vitamin C content in fruits. Vitamin C content peaked on day 5 and then began to decrease again, but the content in the treatment fruits was higher than that in the control fruits. During the storage period, the vitamin C content of treatment group B fruits remained the highest and was 1.09-fold higher than that of the control fruits on day 8 (Figure 8A).

The protein content of fruits in the treatment groups increased during the first 2 days, whereas that of the control groups decreased (Figure 8B), indicating that the fruit protein content was affected by sodium nitroprusside. The protein content decreased from day 3 to day 5, which could be due to biological activity requirements and a series of enzyme reaction consumptions during the ripening process.

## Discussion

Rambutan is a non-climacteric fruit with tropical characteristics, and peel browning, weight loss and firmness reduction are common phenomena in postharvest storage and transportation, being closely related to fruit ripening and ageing (Saba and Moradi, 2017; Hu et al., 2019). Peel browning is associated with water loss, anthocyanin degradation, active oxygen metabolism and enzymatic phenol metabolism (Jiang et al., 2004; Mahajan et al., 2014; Zhang et al., 2015). In this study, the browning index of the four treatment groups increased dramatically from day 5 onwards, indicating excessive water loss from the fruit peel and vigorous fruit metabolism. Postharvest browning negatively affects the quality of the appearance of rambutan fruit, leading to a lack of market competitiveness and significant economic losses (Sivakumar and Korsten, 2010). Therefore, to some extent, sodium nitroprusside could reduce the rate of water loss and regulate metabolism inside the fruit, thereby constraining an increase in the browning index. In addition, sodium nitroprusside has no residual toxicity and is relatively easy to use (Zhu et al., 2008), thereby contributing to its use in commercial methods for postharvest browning control of rambutan fruit.

Biological activities of fruit result in weight loss; however, the weight loss of the rambutan fruits treated with 200 μmol L^−1^ sodium nitroprusside was relatively low, which may be because cell integrity and tissue permeability were more stable under the influence of sodium nitroprusside, and the cell loss was reduced (Koyuncu et al., 2019). Nitric oxide suppresses moisture and weight loss and reduces quality deterioration in banana (Wang et al., 2015), raspberry (Shi et al., 2019), mango (Ren et al., 2017) and other fruits. Therefore, sodium nitroprusside could play an important role in the control of postharvest rambutan fruit loss and quality maintenance. As one of the crucial indicators of appearance quality, fruit firmness is related to mechanical damage and pest infection (Chen et al., 2020). During storage, fruit firmness decreases due to the loss of cell wall hydrolase activity and intracellular turgor pressure, which results in softening (Tokala et al., 2021). In the present study, sodium nitroprusside delayed the softening rate of the rambutan pericarp and maintained fruit firmness, a finding similar to that of Adhikary et al. (2020). Thus, it is likely that sodium nitroprusside contributed to the maintenance of the appearance quality of postharvest rambutans.

Phenolic metabolism and enzymatic phenolic reactions are closely related to peel browning (Tomás-Barberán and Espín, 2001). As an important active substance in fruits and vegetables, the content of phenolic substances is closely related to enzymatic browning and antioxidant capacity (Liu et al., 2002; Kriengsak et al., 2006; Hafiz et al., 2017). Phenolics can scavenge free radicals and delay the lipid oxidation process by suppressing the initiation or diffusion of oxidising chain reactions (Joyce et al., 2004). In our study, soaking fruits in sodium nitroprusside reduced oxidation during storage so that the TPC was maintained at a higher level (Wang et al., 2015), a result in line with the findings of Jiang et al. (2018) and Su et al. (2019). PPO and POD, which are important enzymes related to phenolic metabolism and fruit browning, catalyse oxidation and conversion of phenols into quinones in fruit, thereby causing browning (Sajid et al., 2018). Our findings suggest that 200 μmol L^−1^ sodium nitroprusside may have restrained the activity of PPO and POD, a finding in accordance with the findings of Yingsanga et al. (2008) and Sajid et al. (2019a). The phenolic substances and enzymes were isolated, the synthesis of quinones was reduced, and the integrity of the membrane lipid system was maintained (Xiong, 2016), thereby suppressing the browning process of rambutan peel during storage. Additionally, PAL, an important defence enzyme related to plant disease resistance, participates in the synthesis of phenols related to plant structure and resistance (Zhang et al., 2018b). Moreover, PAL activity is relevant to the production and accumulation of resistant substances, such as phytoalexin and lignin, which are significant indicators of plant resistance to disease and stress (Jiang and Yu, 2001). In this study, the PAL activity of treated fruits increased, a finding analogous to the results of Guo et al. (2013), indicating that these fruits may have been more resistant to pathogen infection, another important factor in postponing fruit decay and ageing. Thus, when treated with sodium nitroprusside, high PAL activity promotes phenol synthesis and the TPC is maintained at a higher level, whereas PPO and POD activities are suppressed, such that higher PAL activity and lower PPO and POD activity lead to the inhibition of phenolic oxidation (Shekari et al., 2021). This is corroborated in other studies (Wang et al., 2010; Zhang et al., 2018a). From the perspective of phenolic metabolism, sodium nitroprusside at 200 μmol L^−1^ may not only have maintained a high phenolic content but may also have reduced oxidation, which plays a key role in the browning of rambutan fruits.

It is universally acknowledged that membrane lipid peroxidation and reactive oxygen species (ROS) are two crucial factors leading to peel browning and quality deterioration of postharvest fruits (Lai et al., 2020). ROS, including O_2_^–•^ and H_2_O_2_, can react with unsaturated fatty acids, triggering oxidative stress and membrane lipid peroxidation (Garg and Manchanda, 2009; Zhang et al., 2018c). MDA is produced by cell lipid peroxidation; furthermore, the MDA content is an indicator of the extent of damage to the body as well as cell ageing (Zhou et al., 2015; Zhang et al., 2020). Oxidative stress destroys the membrane lipid structure and causes cell membrane damage, which leads to increased MDA content in fruits (Sun et al., 2011). Treatment with 200 μmol L^−1^ sodium nitroprusside maintained the lowest MDA content in this study, a finding in line with the results of Lin et al. (2020b) and Shao et al. (2020). Sodium nitroprusside may have inhibited the accumulation of MDA and reduced the MDA content, thereby defending the cell membrane structure from damage (Aghdam et al., 2020) and hindering the lipid peroxidation process to postpone postharvest senescence of the fruit (Masoud et al., 2019). As crucial substances in active oxygen metabolism, oxidative damage mediated by O_2_^–•^ and H_2_O_2_ is a key factor in fruit browning and lipid peroxidation (Duan et al., 2011; Li et al., 2019a). In our study, sodium nitroprusside may have suppressed the production and accumulation of O_2_^–•^ and H_2_O_2_, reduced reactive oxygen damage and exerted a certain delaying and inhibitory effect on browning and senescence (Wang et al., 2015), which was similar to Sajid et al. (2019a, 2019b). Previous studies have shown that the regulation of antioxidant enzyme expression is of great significance to ROS metabolism (Foyer et al., 1994; Lacan and Baccou, 1998). As important components of the antioxidant system (Gao et al., 2016; Aghdam et al., 2020), highly active APX and SOD can reduce the damage to fruit caused by H_2_O_2_ (Zhang et al., 2018a), and the combination of the two can convert ${\text{O}}_{2}^{{-}^{\cdot }}$ into harmless O_2_ and H_2_O in plant tissues (Sajid et al., 2020). In the present study, sodium nitroprusside improved the activity of APX and SOD, potentially activating them to delay the accumulation of ROS and reduce peel damage (Lai et al., 2020) and consequently suppressing browning and senescence (Sajid et al., 2019a). These findings are consistent with the results of Flores et al. (2008) and Shao et al. (2013). Therefore, our findings suggest that the 200 μmol L^−1^ sodium nitroprusside treatment improved the activity of antioxidant enzymes, inhibited the generation of oxidative free radicals, reduced ROS damage and stress and maintained a low MDA content, such that the postharvest browning and senescence of rambutan fruits were controlled.

Through the exploration of phenolic substance metabolism, lipid peroxidation and active oxygen metabolism, the inhibitory effect of sodium nitroprusside on postharvest browning and senescence of rambutans was clarified. In addition, the change in postharvest quality of rambutan fruits was investigated, and it was demonstrated that sodium nitroprusside also plays an important role in retarding fruit quality deterioration.

Total soluble solid and titratable acidity contents are important indicators for fruit quality evaluation and identification and can best represent the taste and flavour of the fruit. It was previously shown that higher TSS content can effectively maintain fruit flavour quality during the storage period (Jiang et al., 2018) and that the maturation and senescence of fruit after harvest will lead to a decrease in TSS content and affect the taste quality (Sajid et al., 2019b). We found that sodium nitroprusside had a delaying effect on the gradual decrease in the fruit TSS content over time. In addition, it is known that the TA has some impact on physiological conditions and quality (Kumar et al., 2020). Sodium nitroprusside at 200 μmol L^−1^ maintained a relatively stable TA content in treated fruits, and the TSS:TA ratio was maintained, so that the fruit flavour was guaranteed.

Vitamin C content in fruits is an important indicator as it affects fruit quality and nutrition (Shao et al., 2013), and changes in vitamin C are related to a variety of factors, such as physiological stress and storage conditions (Asanda et al., 2017). In addition, vitamin C plays a role in scavenging ROS and reducing oxidative damage (Lai et al., 2020). We noted that sodium nitroprusside had a delaying effect on the reduction of vitamin C content, a finding similar to Chaudhary et al. (2015) and Asanda et al. (2017), which may have contributed to the maintenance of fruit quality and postponement of the browning of rambutans. As an important nutrient of rambutan fruit (Lin et al., 2020b), soluble protein content plays a key role in maintaining fruit taste and flavour. Our results showed that the protein content of fruits treated with 200 μmol L^−1^ sodium nitroprusside was maintained at a higher level than that of the other treatments, indicating that this concentration had a better inhibitory effect on the reduction of the protein content. It is known that a higher protein content can delay ripening and senescence of the fruit, as demonstrated by the findings of Jing et al. (2019), Qi et al. (2019) and Zhang (2019). In addition, the changes of sensory quality including flavour and taste of rambutan fruits could be further explored. The contents of glucose, saccharose, fructose would be important and useful for the taste quality. And the occurrence of postharvest diseases is also significantly related to quality deterioration of fruits. According to our previous study (Wang et al., 2021), the control of postharvest pathogens and the defensive ability of plant endophytic fungi against pathogens also have a certain significance in the maintenance of postharvest quality.

## Conclusion

In summary, we found that sodium nitroprusside treatment is an effective and safe method to conserve postharvest quality and delay senescence in rambutan fruits. Application of 200 μmol L^−1^ sodium nitroprusside as a soaking treatment after fruit harvest reduced the rate of browning, inhibited the rate of weight loss and kept fruit firm for longer. Our findings suggest that sodium nitroprusside suppressed weight loss and cell damage, resulting in maintenance of low MDA, ${\text{O}}_{2}^{{-}^{\cdot }}$ and H_2_O_2_ contents; low PPO and POD activity; and high PAL, APX and SOD activity. In addition, sodium nitroprusside maintained favourable quality indicators, such as TA and TSS, vitamin C and protein contents. Our research has demonstrated that nitric oxide derived from sodium nitroprusside has the potential to maintain good postharvest rambutan fruit quality by regulating lipid peroxidation, phenolic metabolism, active oxygen metabolism and the operation of antioxidant systems, thereby contributing to the control of fruit browning and senescence. Therefore, we conclude that sodium nitroprusside soaking can be used as an effective and eco-friendly treatment for the browning control and quality conservation of rambutan fruit during storage. Also, the specific quality indicators and microbiological activities contribute greatly to the postharvest quality and senescence of fruits. In the future work, it is significant to evaluate the eventual effects of the sodium nitroprusside on the quality of the pulp and the temperature is also a critical parameter to extend the shelf life of rambutan fruit.

## Data Availability Statement

The original contributions presented in the study are included in the article.

## Author Contributions

PC and RZ designed the experiment. RZ, ZY, YJ, and FJ implemented the experiment. RZ and ZY processed and analysed the data and prepared the manuscript. RZ and PC revised the manuscript and made modifications. PC supervised the whole study and provided the guidance. All authors have read and approved the manuscript.

## Funding

This research was supported by Hainan Provincial Natural Science Foundation of China (320RC491).

## Conflict of Interest

The authors declare that the research was conducted in the absence of any commercial or financial relationships that could be construed as a potential conflict of interest.

## Publisher’s Note

All claims expressed in this article are solely those of the authors and do not necessarily represent those of their affiliated organizations, or those of the publisher, the editors and the reviewers. Any product that may be evaluated in this article, or claim that may be made by its manufacturer, is not guaranteed or endorsed by the publisher.
